# Seroprevalence of St. Louis Encephalitis Virus and West Nile Virus (*Flavivirus*, *Flaviviridae*) in Horses, Uruguay

**DOI:** 10.1155/2013/582957

**Published:** 2013-12-29

**Authors:** Analía Burgueño, Lorena Spinsanti, Luis Adrián Díaz, María Elisa Rivarola, Juan Arbiza, Marta Contigiani, Adriana Delfraro

**Affiliations:** ^1^Sección Virología, Facultad de Ciencias, Universidad de la República, 11400 Montevideo, Uruguay; ^2^Laboratorio de Arbovirus, Instituto de Virología Dr. J. M. Vanella, Universidad Nacional de Córdoba, 5016 Córdoba, Argentina; ^3^Instituto de Investigaciones Biológicas y Tecnológicas CONICET-FCEFyN, UNC, 5016 Córdoba, Argentina

## Abstract

St. Louis encephalitis virus (SLEV) and West Nile virus (WNV) belong to the Japanese encephalitis antigenic complex (*Flavivirus* genus, *Flaviviridae* family). They show antigenic close relationships and share many similarities in their ecology. Both are responsible for serious human diseases. The aim of this study was to investigate the presence of neutralizing antibodies to these viruses in horses from Uruguay. To do this, 425 horse sera were collected in 2007 and analyzed by plaque reduction neutralization tests. As a result, 205 sera (48.2%) were found positive for SLEV, with titers ranging between 10 and 80. Two sera remained inconclusive, since they showed low titers to WNV and SLEV (10 and 20), not allowing us to demonstrate activity of WNV in our territory. This is the first report of circulation of SLEV in horses in Uruguay.

## 1. Introduction

Infections caused by mosquito-borne flaviviruses are among the diseases with higher incidence in the world. Most of them are asymptomatic or present an influenza-like illness; however severe cases may occur, causing central nervous system disease, coma, and/or death [1, 2].

St. Louis encephalitis virus (SLEV) and West Nile virus (WNV) are members of the Japanese encephalitis antigenic complex; they are usually maintained in cycles between birds and *Culex* mosquitoes; humans and horses are dead-end hosts. These viruses can cause encephalitis in humans; only WNV infection may lead to fatal encephalitis in equines. All flaviviruses are antigenically closely related, giving frequent serological cross-reactions which are exaggerated in sequential infections. Because of this, specific etiologic diagnosis may be difficult specially in areas where two or more flaviviruses are prevalent [3].

SLEV is widely distributed in the Americas. Recently, human outbreaks were reported in Argentina in 2005 and 2010 [4, 5] and in Brazil in 2006 [6]. Regarding arbovirus circulation in Uruguay, the first reports of SLEV date from the 70s and correspond to serological studies in children and adults, with 4% and 5% seroprevalence, respectively [7]. In 1997 the Ministry of Public Health of Uruguay began dengue virus surveillance through serological diagnosis in human cases and detection and control of the vector *Stegomyia (Aedes) aegypti*. Until today, only imported cases were diagnosed but the vector is present in several departments of the country, reinforcing the need of a close surveillance of this flavivirus [8]. More recently, in 2001, as part of dengue surveillance, a cluster of febrile illness cases were confirmed as SLEV infections [9]. In 2010 the Ministry of Public Health initiated the monitoring of viral meningoencephalitis; since then 3 cases of SLEV were serologically confirmed, all in 2012 [10].

WNV was introduced in the Americas in 1999, in the city of New York, United States [11]. Since then, the virus has moved to the south of the continent, with reports of infections in birds and equines in the Caribbean, Venezuela, Colombia, and Argentina [12–16]. No records of cases or data are available for WNV in Uruguay.

The objective of the present study was to analyze the presence of neutralizing antibodies to SLEV and WNV in equines, in order to investigate the circulation of these flaviviruses in Uruguay.

## 2. Materials and Methods

### 2.1. Study Sites

**Figure 1:**
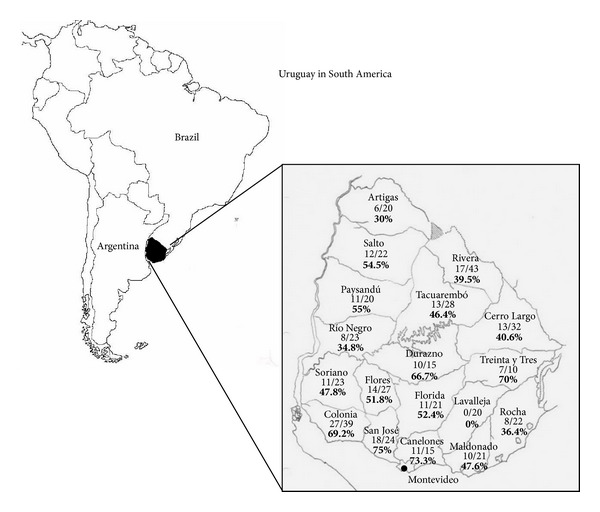
Map of Uruguay in South America, showing location of sampling sites, number of positive/number of horses sampled per department, and seroprevalence percentage (in boldface).

Uruguay, located in the south cone of South America, has a total surface of 176.215 km^2^ and a population of 3.356.584 inhabitants. It is divided in 19 departments (Figure 1) and the local weather is temperate (average: 17.5°C) and humid (average: 75%) with homogeneous rainfall throughout the year. The economy is based on agriculture and livestock activities.

### 2.2. Sample Collection

During 2007, 425 horse serum samples were collected by the Veterinary Laboratories Division (DI.LA.VE.) “Miguel C. Rubino” (Ministry of Agriculture and Fisheries) to perform epidemiologic studies. None of the horses presented any sign of disease. Blood samples were taken from the jugular vein of animals and kept at −20°C until processed. To do the present study, a minimum of 10 sera per department were tested, belonging to 18 of the 19 departments (Figure 1). Samples from Montevideo department were not available.

### 2.3. Plaque Reduction Neutralization Test (PRNT)

Detection of neutralizing antibodies to SLEV and WNV was performed using plaque reduction neutralization tests (PRNT) in VERO cells as previously described [17]. SLEV strain 78V6507 [18] and WNV strain E/7229/06 [15] were used. Screening of sera was done at 1 : 10 dilution and endpoints were determined at 80% (PRNT_80_). Positive samples were further titrated in serial dilutions beginning at 1 : 20.

## 3. Results

In the screening test, 207 (48.7%) of the 425 horses had neutralizing antibodies to SLEV. Positive samples were found in 17 of the 18 departments tested (all departments except for Lavalleja) (Table 1). By department, seroprevalence ranged between 0 and 75%, with the highest value corresponding to San José (18 positives from 24 tested). PRNT_80_ titers of SLEV seropositive horse sera ranged from 10 to 160 (data available under request).

Of the 207 SLEV seropositive sera found in the screening, 90 were further titrated in a simultaneous test for both SLEV and WNV. No seropositive sera to WNV were detected at this stage. In view of this result, and taking into account that PRNT is a highly time and resource consuming technique, we decided not to continue with the simultaneous titration. So, all other SLEV seropositive samples in the screening (*n* = 117) were in turn screened to WNV. After these assays, eight sera were positive for WNV at a 1 : 10 dilution. These eight sera were in turn simultaneously titrated to SLEV and WNV. As result, six sera showed a fourfold or greater difference in titer to SLEV in respect to WNV and two sera remained inconclusive (Table 2). Finally, a total of 205 samples (48.2%) were considered as SLEV positive.

## 4. Discussion

We report the first evidence of neutralizing antibodies to SLEV in horses in Uruguay. SLEV transmission appears to be widely distributed in the country and has no geographic restrictions, since we found antibodies in sera from 17 of the 18 departments analyzed.

Our studies indicated a 48.2% seroprevalence for SLEV, similar to previously reported values in neighbor countries. In Argentina, Monath et al. found SLEV seroprevalence ranging from 42 to 75% [19]. More recently a study of Tauro et al. in Santa Fe province found a 12% seroprevalence, much lower than the one found by Monath. As the authors stated in their article, this seroprevalence could be underestimated due to the use of an alternative (more stringent) criterion of positivity [20].

In Brazil, Rodrigues et al. investigated the immunity of horses against SLEV in Mato Grosso do Sul State and the Brazilian Amazon region, and they found a seroprevalence of 50.9%. In turn, Pauvolid-Corrêa et al. found a seroprevalence of 43.7% in horses from the Nhecolândia subregion in South Pantanal (Central-West Brazil) [21, 22].

To minimize the cross-reaction results in this survey, we followed the Centers for Disease Control and Prevention's (CDC) guidelines for seropositivity: when comparing sera titers to two or more viruses, a fourfold difference in PRNT is needed to identify the etiologic agent. According to this criterion, the simultaneous titration resulted in six sera seropositive to SLEV and two inconclusive. From these inconclusive sera, sample E07/204 showed a greater titer to WNV (20) than SLEV (10). These results could be due to an initial infection with WNV followed by a secondary infection with SLEV. Patiris et al. demonstrated in chickens that sequential infections with SLEV (1st) and WNV (2nd) greatly amplified antibodies to SLEV. The reverse order of infection (1st infection with WNV and 2nd infection with SLEV) produced a limited response, and titers to both viruses remained similar. In turn, titers to WNV may be considered as result of a very recent infection or a cross-reaction for an undetermined flavivirus. Lederman et al. showed that after an initial exposition to SLEV followed by a subsequent infection with WNV, detectable levels of WNV specific antibodies developed at days 9–12, with a peak at days 12–18 [23, 24].

It is noteworthy that, a year before our sampling, WNV virus was actively circulating in the region. In 2006, the virus was isolated for the first time in South America from sick horses in Argentina, and evidence of WNV activity in birds was demonstrated in Córdoba province (Argentina) in 2005 and 2006 [12, 15]. The inconclusive results regarding WNV activity reinforce the need of further studies to confirm or discard its circulation in Uruguay.

SLEV activity in horses described in this study, together with the reports of sporadic human infections and the diagnosis of three cases of SLEV meningoencephalitis (two in Montevideo and one in San José department), confirm the circulation of this virus in our country [9, 10]. Our study shows that the highest seroprevalence corresponded to San José and Canelones departments (75% and 73.3%, resp.). Although we have no data from Montevideo, it is noteworthy that San José and Canelones are bordering departments. In addition, 59% of the Uruguayan population resides in these departments, so this Southern area of the country may be at risk for future outbreaks. In turn, the relatively low number of SLEV human cases in comparison with the high seroprevalence found in horses could be due to unapparent infections or the lack of febrile syndrome surveillance. Our results highlight the need for initiating the surveillance of febrile syndromes and sustaining the monitoring of viral encephalitis in Uruguay.

Regarding arbovirus investigation in Uruguay, our lab is conducting since 2007 the molecular detection in mosquitoes. Although we still did not detect SLEV in the vectors, it is to note that *Culex *spp. represents the 80% of mosquitoes captured in our studies, so risk of outbreaks should not be discarded.

## 5. Conclusions

The results presented here show that SLEV is actively circulating in our country at a significant prevalence (48.2%). Seroprevalences ranged between 0 and 75%; San José and Canelones departments presented the highest values (75% and 73.3%, resp.). Our results do not demonstrate the circulation of WNV in Uruguay; however recent infections with this virus or circulation of another flaviviruses cannot be excluded.

Our findings, together with the sporadic SLEV infections documented in humans and the risk of introduction of dengue virus in our territory, reinforce the need of differential diagnosis of flaviviruses. With the aim of contributing to the knowledge of the ecology of flavivirus in our country, our research group is carrying out arbovirus detection in mosquitoes and initiating serologic studies in wild birds.

## Figures and Tables

**Table 1:** Results of the PRNT_80_ screening to SLEV and WNV.

Department	No. of samples analyzed	No. of SLEV positive	No. of WNV positive*
Artigas	20	6	0
Canelones	15	11	2
Cerro Largo	32	13	0
Colonia	39	27	1
Durazno	15	10	0
Flores	27	14	0
Florida	21	11	1
Lavalleja	20	0	0
Maldonado	21	10	0
Paysandú	20	11	1
Río Negro	23	8	0
Rivera	43	17	0
Rocha	22	8	0
Salto	22	12	1
San José	24	18	0
Soriano	23	11	1
Tacuarembó	28	13	0
Treinta y Tres	10	7	1

Total	425	207	8

*Samples positive both to WNV and SLEV.

**Table 2:** PRNT_80_ simultaneous titration to SLEV and WNV.

Sample	Department	Antibody titer		Result
		SLEV	WNV	
E07/204	Salto	10	20	Inconclusive
E07/210	Canelones	80	10	SLEV
E07/211	Colonia	40	10	SLEV
E07/245	Paysandú	80	10	SLEV
E07/274	Florida	10	10	Inconclusive
E07/287	Canelones	80	10	SLEV
E07/408	Soriano	80	20	SLEV
E07/421	Treinta y Tres	40	10	SLEV
